# CD4^+^CD62L^+^ Central Memory T Cells Can Be Converted to Foxp3^+^ T Cells

**DOI:** 10.1371/journal.pone.0077322

**Published:** 2013-10-14

**Authors:** Xiaolong Zhang, Xian Chang Li, Xiang Xiao, Rui Sun, Zhigang Tian, Haiming Wei

**Affiliations:** 1 Institute of Immunology, School of Life Sciences, University of Science & Technology of China, Hefei, Anhui, China; 2 Transplant Research Center, Brigham and Women's Hospital & Children's Hospital Boston, Harvard Medical School, Boston, Massachusetts, United States of America; 3 Hefei National Laboratory for Physical Sciences at Microscale, University of Science and Technology of China, Hefei, Anhui, China; MRC National Institute for Medical Research, United Kingdom

## Abstract

The peripheral Foxp3^+^ Treg pool consists of naturally arising Treg (nTreg) and adaptive Treg cells (iTreg). It is well known that naive CD4^+^ T cells can be readily converted to Foxp3^+^ iTreg *in vitro*, and memory CD4^+^ T cells are resistant to conversion. In this study, we investigated the induction of Foxp3^+^ T cells from various CD4^+^ T-cell subsets in human peripheral blood. Though naive CD4^+^ T cells were readily converted to Foxp3^+^ T cells with TGF-β and IL-2 treatment *in vitro*, such Foxp3^+^ T cells did not express the memory marker CD45RO as do Foxp3^+^ T cells induced in the peripheral blood of Hepatitis B Virus (HBV) patients. Interestingly, a subset of human memory CD4^+^ T cells, defined as CD62L^+^ central memory T cells, could be induced by TGF-β to differentiate into Foxp3^+^ T cells. It is well known that Foxp3^+^ T cells derived from human CD4^+^CD25^-^ T cells *in vitro* are lack suppressive functions. Our data about the suppressive functions of CD4^+^CD62L^+^ central memory T cell-derived Foxp3^+^ T cells support this conception, and an epigenetic analysis of these cells showed a similar methylation pattern in the FOXP3 Treg-specific demethylated region as the naive CD4^+^ T cell-derived Foxp3^+^ T cells. But further research showed that mouse CD4^+^ central memory T cells also could be induced to differentiate into Foxp3^+^ T cells, such Foxp3^+^ T cells could suppress the proliferation of effector T cells. Thus, our study identified CD4^+^CD62L^+^ central memory T cells as a novel potential source of iTreg.

## Introduction

Regulatory T cells play an important role in self-tolerance, acquired tolerance, and immunological homeostasis [1,2]. There are multiple types of immune regulatory T cells, including Tr1 cells, natural killer T cells, CD8^+^ T cells and CD4^+^CD25^+^Foxp3^+^ cells [3]. CD4^+^CD25^+^Foxp3^+^ cells (referred to as Treg) are the predominant regulatory T cells. Treg, which are defined by their expression of Foxp3, are broadly subdivided into nTreg and iTreg [4]. nTreg are generated by the interactions between thymic T cell receptors (TCRs) with a high affinity for MHC class II ligands in the thymus. These cells help to maintain tolerance to self-antigens to prevent autoimmunity and to regulate immune responses by raising activation thresholds. Induced Treg cells are potentially derived from various conditions outside the thymus, a phenomenon that has been supported by numerous *in vivo* studies [4]. 

 Although it is clear that iTreg are converted from activated T effector cells in the periphery, the origin of activated T effector cells is unclear. Previous *in vivo* evidence has suggested that iTreg are derived from conventional CD4^+^CD25^-^ T cells in the periphery [5-7]. Further *in vitro* studies supported this notion, as iTreg can be efficiently differentiated from purified CD4^+^CD25^-^ T cells via TGF-β stimulation [8]. Recently, other studies reported that of the CD4^+^CD25^-^ T cells, only naive CD4^+^ T cells (Tn) but not memory CD4^+^ T cells (Tm) are able to differentiate into iTreg *in vitro* in both mouse and human models [9-12]. It is generally accepted that iTreg are converted from activated naive CD4^+^CD25^-^ T cells *in vivo*. Although naive CD4^+^CD25^--^ T cells can be converted into iTreg by TGF-β, the ability of memory CD4^+^CD25^--^ T cells to differentiate into iTreg remains controversial. One report has shown that human Foxp3^+^ iTreg are derived from the rapid turnover of peripheral memory CD4^+^Foxp3^-^ T cells *in vivo*, and another found that mouse Th2 memory T cells can be efficiently converted into Foxp3^+^ iTreg *in vitro* using a differentiation protocol [13,14]. Additionally, human skin-derived memory Th cells can be converted into Foxp3^+^ iTreg with a suitable manipulation [15]. These studies revealed the possibility that memory CD4^+^CD25^--^ T cells can differentiate into iTreg. As human memory CD4^+^CD25^--^ T cells are not a uniform population (like naive CD4^+^CD25^-^ T cells) [16], various subsets of memory CD4^+^ T cells may have different capabilities for differentiating into iTreg. Thus, we cannot exclude the possibility that iTreg are converted from activated memory CD4^+^CD25^-^ T cells *in vivo*. Because iTreg play essential roles in the development of immune tolerance to foreign antigens and tumour-derived neo-antigens [17,18], a better understanding of their origin and differentiation would provide important information regarding their therapeutic potential.

Here, we showed that human memory CD4^+^CD25^-^ T cells could be efficiently differentiated into Foxp3^+^ T cells via TCR activation with TGF-β, but this phenomenon was restricted to CD62L^+^CCR7^+^ memory CD4^+^ T cells. Furthermore, we also found this phenomenon in mice and proved that such CD4^+^CD62L^+^ central memory T cell-derived Foxp3^+^ T cells were functional in suppressing the proliferation of T effector cells. Our data suggest that CD4^+^CD62L^+^ central memory T cells can be differentiated into functional Foxp3^+^ iTreg, at least in mice. These findings may have important implications for understanding the development of iTreg *in vivo*.

## Materials and Methods

### Ethics Statement

Healthy human peripheral blood was obtained from the Blood Centre of Anhui Province (Hefei, China). Peripheral blood was obtained from chronic HBV-infected and healthy women at the Centre for Disease Control and Prevention in Hefei, China. Ethical approval to use these samples was obtained from the Ethics Committee of the University of Science and Technology of China (Permit Number: USTCACUC1201011). In accordance with the Declaration of Helsinki and the Institutional Review Board of the University of Science and Technology of China, all participants provided written informed consent, which was obtained before enrolment in the study.

The study was carried out in strict accordance with the recommendations in the Guide for the Care and Use of Laboratory Animals. A GFP-Foxp3 fusion protein was created by introducing a bicistronic enhanced GFP reporter gene into the endogenous Foxp3 loci of B6 mice (foxp3^gfp^), as previously reported [19]. These knock-in mice were bred and maintained at Harvard Medical School in Boston. All protocols involving animal work were approved by the Animal Care and Use Committee at Harvard Medical School (Permit Number: 004848). All surgery was performed under anaesthesia, and all efforts were made to minimize animal suffering.

### Cell purification

Peripheral blood mononuclear cells (PBMCs) were prepared by centrifugation over Ficoll-Hypaque gradients and then stained with APC-Cy7-anti-CD4, PE-anti-CD25, FITC-anti-CD45RA, and APC-anti-CD45RO (or APC-Cy7-anti-CD4, PE-anti-CD25, FITC-anti-CD62L, APC-anti-CD45RO, PE-Cy7-anti-CCR7) for 30 min at 4°C. The CD45RA^+^CD45RO^-^ (naive) and CD45RA^-^CD45RO^+^ (memory) cells within the CD4^+^CD25^-^ family were gated and selectively sorted with a FACSAria flow cytometer. CD62L^+^CCR7^+^ naive, CD62L^+^CCR7^+^ memory, CD62L^+^CCR7^-^ memory, and CD62L^-^CCR7^-^ memory cells within the CD4^+^CD25^-^ family were obtained as above. For the isolation of naive, central memory, and effector memory CD4 T cells from Foxp3^gfp^ mice, mouse spleen cells were prepared and stained with PE-Cy5-anti-CD4, PE-Cy7-anti-CD44, and Pacific Blue-anti-CD62L for 20 min at 4°C. The CD44^low^CD62L^high^ cells (naive), CD44^high^CD62L^high^ cells (central memory) and CD44^high^CD62L^low^ cells (effector memory) within the CD4^+^ GFP (Foxp3)^-^ family were gated and selectively sorted. The purity of sorted cells was higher than 95%. The antibodies used above were all from BD Bioscience (SanJose, CA)

### Conversion of CD4^+^Foxp3^-^ T cells into Foxp3^+^ T cells in vitro

Peripheral blood mononuclear cells (PBMCs) were prepared by centrifugation over Ficoll-Hypaque gradients from healthy human peripheral blood. Naive, memory, central memory and effector memory human CD4 T cells were sorted from the prepared PBMCs. The FACS-sorted human CD4 T cells were stimulated *in vitro* (5×10^4^ cells/well) with plate-bound anti-CD3 (5 μg/ml; BD Bioscience) and soluble anti-CD28 (1 μg/ml; BD Bioscience) for 1–7 days in the presence of recombinant human TGF-β (5 ng/ml; R&D) and IL-2 (100 U/ml; Peptech) or not. The induction of Foxp3^+^ T cells in the CD4^+^ fraction was analysed by FACS based on the intracellular staining of the Foxp3 protein. FACS-sorted mouse naive, central memory, and effector memory CD4 T cells were stimulated *in vitro* (1×10^5^ cells/well) with anti-CD3 (2 μg/ml; BD Pharmingen) and APCs (1×10^5^ cells/well) in the presence of recombinant TGF-β (3 ng/ml; R&D) and IL-2 (100 U/ml; Peptech) for 3–5 days. APCs were obtained from syngeneic mice by depleting T cells from a population of spleen cells and then treating them with mitomycin C (50 μg/ml; Sigma-Aldrich) at 37°C for 20 min. The induction of Foxp3^+^ T cells in the CD4^+^ fraction was analysed by FACS based on the expression of GFP.

### Flow cytometric analysis

For intracellular Foxp3 and CTLA-4 staining, cultured cells were resuspended and labelled with the fluorochrome-conjugated specific mAbs against surface markers for 30 min at 4°C. The cells were then fixed and permeabilised using the Fixation/Permeabilization intracellular staining kit according to the manufacturer’s protocol (eBioscience). After fixation and permeabilisation, cells were incubated with Percp-cy5.5-anti-FOXP3 (eBioscience) and PE-anti-CTLA-4 (BD Bioscience) Abs. Data were acquired with a FACSCalibur flow cytometer (BD Bioscience) and analysed with FlowJo software (Tree Star).

### Suppression assays in vitro

Human naive CD4^+^ T cells, CD62L^+^CCR7^+^ central memory CD4^+^ cells, and CD62L^-^CCR7^-^ effector memory CD4^+^ cells were stimulated *in vitro* (5×10^4^ cells/well) for 5 days in the presence of TGF-β and IL-2. After stimulation, the cells were harvested, rested for 2 days in IL-2-containing culture medium and then washed three times with PBS prior to the suppression assay. These cells were used as suppressor cells. CD4^+^CD25^-^ T cells, CD4^+^CD25^high^ Treg cells and CD3^-^ PBMCs were purified from healthy human PBMCs. CD4^+^CD25^-^ T cells labelled with 5 mM CFSE (Invitrogen) were used as responder T cells (T_*eff*_), and irradiated CD3^-^ PBMCs (3000 rad) were used as antigen-presenting cells (APCs) in suppression assays. The labelled T cells (2.5×10^4^ cells/well) were co-cultured with varying numbers of CD4^+^CD25^high^ Treg or induced Foxp3^+^ T cells to achieve the desired Treg/T_*eff*_ cell ratios. The cells were then stimulated *in vitro* either with or without APCs (5×10^4^ cells/well) prior to stimulation with 5 μg/mL anti-CD3 and with or without 1 μg/mL anti-CD28 in 96-well culture plates in supplemented RPMI medium for 5 days. Responder T cell proliferation with or without induced Foxp3^+^ T cells was assessed using flow cytometric CFSE dilution after 5 days.

CD4^+^GFP (Foxp3^-^) T cells were sorted from Foxp3^gfp^ mice, labelled with CFSE and used as responder cells. The labelled T cells (1×10^5^ cells/well) were mixed with varying numbers of GFP^+^ (Foxp3^+^) T cells to achieve the desired Treg/T effector cell ratios. CD4^+^GFP^+^ (Foxp3^+^) T cells were sorted from naive or central memory CD4^+^ T cells through the induction of Foxp3. The cell mixture was stimulated with syngeneic APCs (1×10^5^ cells/well) and anti-CD3 (1 μg/ml). The T cell-depleted spleen cells were briefly treated with mitomycin C and used as APCs. After 3 days, responder T cell proliferation with or without Foxp3^+^ T cells was assessed using flow cytometry based on the dilution of the CFSE dye. 

### FOXP3 Treg-specific demethylated region (TSDR) DNA methylation analysis

Cells for this series of experiments were from normal male donor peripheral blood to avoid the problems with interpretation from X-chromosomal inactivation of one FOXP3 allele in the methylation analysis. FACS-sorted naive and CD62L^+^ central memory CD4^+^ cells were stimulated for 5 days in the presence of IL-2 (100 U/ml) with or without TGF-β. Sorted CD4^+^CD25^high^ Tregs were stimulated for 5 days in the presence of IL-2 (300 U/ml) as a control. Genomic DNA of cultured cells was extracted by using the EasyPure^TM^ Genomic DNA kit (TransGen Biotech) according to the manufacturer’s protocol for cultured mammalian cells. Bisulfite conversion of genomic DNA was performed by the EZ DNA Methylation-Gold™ Kit (ZYMO RESEARCH) according to the manufacturer’s instructions. DNA was then subjected to PCR with the following TSDR-primers (5’ to 3’ direction): forward –TGTTTGGGGGTAGAGGATTT and reverse –TATCACCCCACCTAAACCAA [20]. PCR was performed in a final volume of 25 μl containing 1x PCR Buffer, 1.25 U Taq DNA polymerase (TaKaRa, LA Taq Hot Start Version), 200 mM dNTPs, 100 pmol each of forward and reverse primers, and 2 μl of bisulfite-treated genomic DNA at 95°C for 10 min and 40 cycles of 95°C for 1 min, 55°C for 45 sec and 72°C for 1 min with a final extension step of 10 min at 72°C. PCR products were purified using the Wizard SV Gel and PCR Clean-Up System (Promega). The purified PCR products were then cloned into the pMD19-T vector (TaKaRa), and 15 individual clones from each sample were cycle sequenced by the ABI 3730 genetic analyzer (Applied Biosystems). DNA methylation analysis and diagram generation were performed by BiQ Analyzer [21].

### Statistical analysis

GraphPad Prism 5.00 software (GraphPad) was used to compare the difference among different groups by using the unpaired *t* test, when values at p<0.05 were considered as significant.

## Results

### The surface expression of CD45RO distinguishes between human induced CD4^+^Foxp3^+^ T cells populations in vitro and in vivo

**Figure 1:**
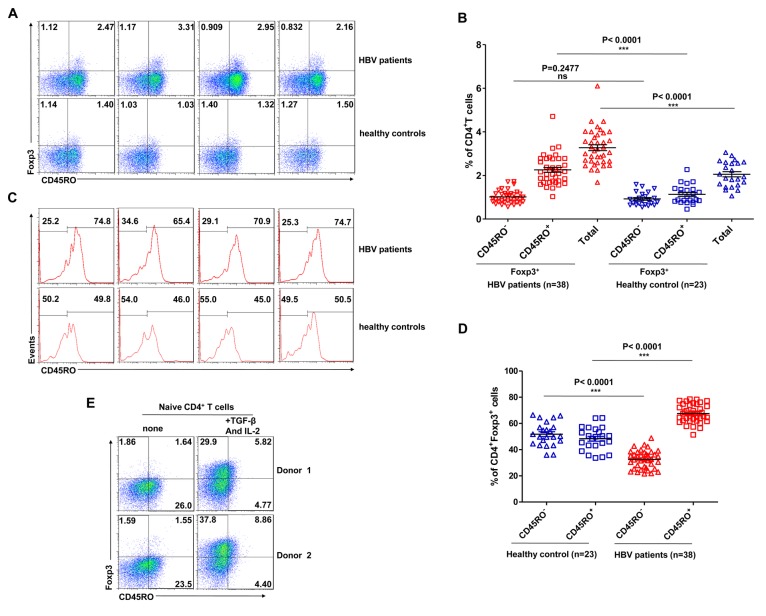
Differential CD45RO expression between Foxp3^+^ T cells differentiated *in* *vivo* by chronic viral infection and *in* *vitro* by TCR stimulation. (A) Flow cytometric analysis of Foxp3 expression by CD4^+^ PBMCs of 4 HBV-infected patients (upper panels) and 4 healthy controls (lower panels). The frequency of cells in each quadrant is displayed as numbers in the quadrants. (B) The average percentages of CD45RO^+^Foxp3^+^, CD45RO^-^Foxp3^+^ and Foxp3^+^ cells in the pools of CD4^+^ cells from 38 HBV-infected patients and 23 healthy controls. (C) Flow cytometric analysis of CD45RO expression by CD4^+^Foxp3^+^ PBMCs of 4 HBV-infected patients (upper panels) and 4 healthy controls (lower panels). The frequency of cells in each quadrant cells is displayed as numbers in the quadrants. (D) The average percentages of CD45RO^+^ and CD45RO^-^ cells among pools of CD4^+^Foxp3^+^ cells from 38 HBV-infected patients and 23 healthy controls. (E) Naive CD4^+^ T cells were sorted from adult peripheral blood by FACS, and the cells were activated with plate-bound anti-CD3 (5 μg/ml) and soluble anti-CD28 (1 μg/ml) in the absence or presence of TGF-β (5 ng/ml) and IL-2 (100 UI/ml) for 5 days. The cells were then collected and analysed by flow cytometry to evaluate the expression of Foxp3 and CD45RO.

Human CD4^+^Foxp3^+^ T cells are phenotypically heterogeneous and include the CD45RO^+^ and CD45RA^+^ T cell subtypes [22,23]. To examine the phenotype of CD4^+^Foxp3^+^ T cells induced *in vivo*, PBMCs from chronic HBV-infected women in the inactive (IN) phase (n=38) and from healthy women (n=22) were analysed by flow cytometry, detailed information on the patient’s ages are shown in Table S1. We first gated on CD4^+^ cells from the PBMCs (Figure 1A) and found that the proportion of CD4^+^Foxp3^+^ T cells was significantly higher in chronic HBV-infected women than in the healthy controls (3.271±0.1394 vs. 2.055±0.1200; P<0.0001) (Figure 1B). We also observed a significantly higher proportion of CD45RO^+^Foxp3^+^ T cells in the PBMCs of chronic HBV-infected women compared to the healthy controls (2.257±0.1111 vs. 1.130±0.08875; P<0.0001) (Figure 1B). We then gated on CD4^+^Foxp3^+^ cells from the PBMCs (Figure 1C) and further confirmed a significantly higher proportion of CD45RO^+^ cells in the CD4^+^Foxp3^+^ cells of chronic HBV-infected women compared to the healthy controls (67.49±1.117 vs. 48.27±1.857; P<0.0001) (Figure 1D). These results suggested that the induced Foxp3^+^ T cells in the peripheral blood of HBV patients expressed the memory marker CD45RO.

We further examined whether CD4^+^Foxp3^+^ T cells induced *in vitro* had the same CD45RO expression as CD4^+^Foxp3^+^ T cells induced *in vivo*. We purified CD4^+^ naive T cells from human PBMCs and induced CD4^+^Foxp3^+^ by T-cell receptor (TCR) activation *in vitro*. We found that the CD4^+^ naive T cells that were activated either by TCR stimulation alone or together with TGF-β and IL-2 rarely co-expressed CD45RO and Foxp3 (Figure 1E). When we analysed the expression of CD45RA, we found that naive CD4^+^ T cells showed no CD45RA expression under stimulation *in vitro* (Figure S1).

The above results collectively show that CD4^+^Foxp3^+^ T cells generated *in vitro* through TCR stimulation express different levels of CD45RO than *in vivo* chronic virus infection-induced CD4^+^Foxp3^+^ T cells, which suggests that CD4^+^Foxp3^+^ T cells induced *in vivo* might not all be derived from CD4^+^ naive T cells.

### A small portion of human CD4^+^ memory T cells can differentiate into Foxp3^+^ T cells by TGF-β stimulation

**Figure 2:**
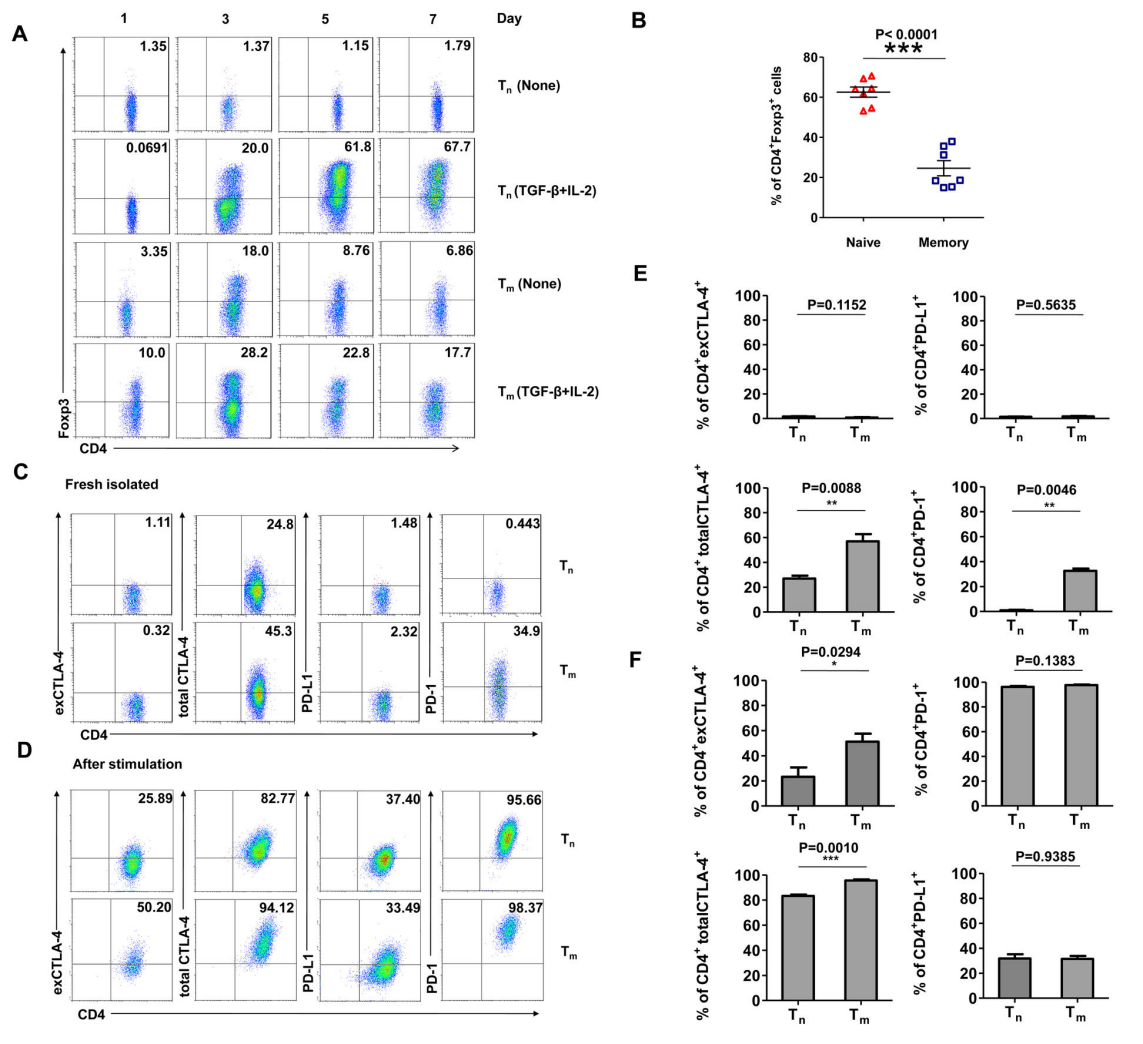
Small portion of human CD4^+^ memory T cells can be converted to Foxp3^+^ T cells *in* *vitro*. (A) CD4^+^ naive (Tn) and memory (Tm) T cells were sorted and activated with plate-bound anti-CD3 (5 μg/ml) and soluble anti-CD28 (1 μg/ml) in the presence or absence of TGF-β (5 ng/ml) and IL-2 (100 UI/ml). Foxp3 expression was analysed by flow cytometry at different time points as indicated. (B) The average percentages of Foxp3 expression in CD4^+^ naive and memory T cells on day 5 after TGF-β and IL-2 induction. (C) and (E) Flow cytometric analysis of CTLA-4 (extracellular and total), PD-1, and PD-L1 expression in total, freshly sorted, naive (Tn) or memory (Tm) CD4^+^ T cells. (D) and (F) Sorted CD4^+^ naive (Tn) or memory (Tm) T cells were activated for 5 days as described above, and the total population was analysed using flow cytometry for the expression of CTLA-4 (extracellular and total), PD-1, and PD-L1.

CD4^+^ naive (Tn) and memory (Tm) T cells from healthy human PBMCs were sorted using flow cytometry and investigated for their *in vitro* induction of Foxp3 in the presence or absence of TGF-β/IL-2 (Figure 2A). Consistent with previous studies in human and mouse CD4^+^ T cells, we showed that TGF-β-mediated Foxp3 expression was significantly higher in CD4^+^ naive T cells than in CD4^+^ memory T cells. However, a small undefined population of CD4^+^ memory T cells was induced to express Foxp3 (Figure 2B).

Treg express several inhibitory molecules, such as CTLA-4, PD-1, and PD-L1, which are potentially necessary for their suppressive functions [24-26]. We analysed the expression of these molecules by flow cytometry before and after stimulation with TGF-β. Prior to stimulation, the expression levels of membrane CTLA-4 and PD-L1 were not significantly different between CD4^+^ naive and memory T cells. However, PD-1 was expressed in CD4^+^ memory but not naive T cells. In addition, both CD4^+^ naive and memory T cells expressed intracellular CTLA-4, but the expression level was significantly higher in memory T cells (Figure 2C,E). After stimulation, the CD4^+^ memory T cells cultured with TGF-β expressed higher levels of membrane CTLA-4 than CD4^+^ naive T cells. The total cytosolic and membrane CTLA-4 expression levels were similar between the cell populations. The expression levels of PD-L1 and PD-1 were also similar between the two cell populations (Figure 2D,F).

The above results showed that, although CD4^+^ memory T cells were resistant to TGF-β-mediated Foxp3 expression, they expressed higher levels of membrane CTLA-4 than CD4^+^ naive T cells. The high level of membrane CTLA-4 expression in memory CD4^+^ T cells that is observed upon Foxp3 induction and the fact that some CD4^+^ memory T cells can be induced to express Foxp3 suggest that a special subset of CD4^+^ memory T cells might have the ability to differentiate into Foxp3^+^ T cells. Thus, we next explored the ability of different subsets of CD4^+^ memory T cells to differentiate into Foxp3-expressing T cells.

### CD4^+^CD62L^+^ central memory T cells can differentiate into Foxp3^+^ T cells with TGF-β treatment

**Figure 3:**
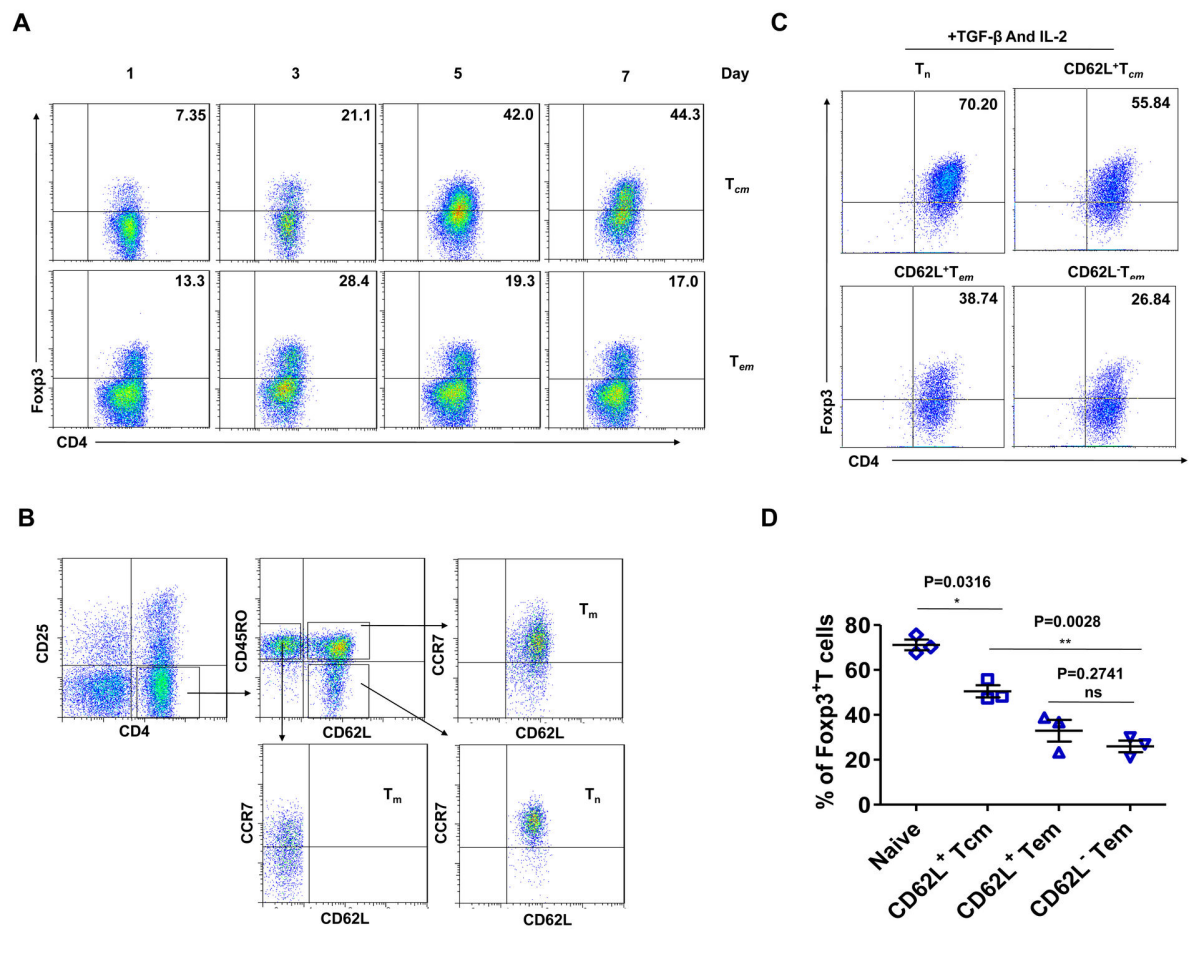
More efficient induction of Foxp3^+^ T cells by CD4^+^CD62L^+^ central memory T cells than by other memory T cell subsets. (A) Sorted CD4^+^ central memory and effector memory T cells were activated with plate-bound anti-CD3 (5 μg/ml) and soluble anti-CD28 (1 μg/ml) in the presence of TGF-β (5 ng/ml) and IL-2 (100 UI/ml) for 7 days. The induction of Foxp3^+^ T cells in the CD4^+^ fraction was analysed by flow cytometry based on the intracellular staining of Foxp3. (B-D) Tn indicates sorted CD4^+^ naive T cells, CD62L^+^ T_*cm*_ indicates sorted CD4^+^CD62L^+^ central memory T cells, CD62L^+^ T_*em*_ indicates sorted CD4^+^CD62L^+^ effector memory T cells, and CD62L^-^ T_*em*_ indicates sorted CD4^+^CD62L^-^ effector memory T cells. The results are one representative of three independent experiments. (B) Flow cytometric analyses of the CD4^+^ naive and memory T-cell phenotypes. (C) Sorted CD4^+^ naive, CD62L^+^ central memory, CD62L^+^ effector memory and CD62L^-^ effector memory T cells were activated for 5 days as above and analysed by flow cytometry for Foxp3 expression in the indicated group of cells. (D) The average percentages of Foxp3 expression by different subsets of memory T cells after induction by TGF-β and IL-2.

Human memory T cells can be divided into central memory and effector memory cells based on CCR7 expression. Thus, we sorted human CD4^+^ memory T cells into CCR7^+^ (central memory, T_*cm*_) and CCR7^-^ (effector memory, T_*em*_) T cells and compared their responsiveness to TGF-β stimulation. The results showed that the differentiation efficiency of central memory T cells was higher than that of effector memory T cells (Figure 3A). Although the CD4^+^ central memory T cells were induced into Foxp3^+^ T cells more efficiently than the effector memory T cells, the rate of differentiation was still lower than that of CD4^+^ naive T cells. This led us to perform a more detailed surface marker analysis of the CD4^+^ central memory T cell pool. This analysis identified a group of cells that were doubly positive for CD62L and CCR7 and had a similar surface expression phenotype to CD4^+^ naive T cells (Figure 3B). Because CD4^+^CD45RO^-^ T cells express a high level of CD45RA (Figure S2), we considered CD4^+^CD45RO^-^ T cells to be CD4^+^CD45RA^+^ naive T cells. We then isolated these groups of CD4^+^ memory T cells based on CD62L and CCR7 expression and examined their response to TGF-β induction. As expected, the differentiation efficiency of CD4^+^CD62L^+^ central memory T cells was close to that of CD4^+^ naive T cells, while the other types of CD4^+^ memory T cells differentiated at low rates (Figure 3, C and D).

### Foxp3^+^ T cells derived from human CD4^+^CD62L^+^ central memory cells in vitro do not exhibit apparent suppressive functions

**Figure 4:**
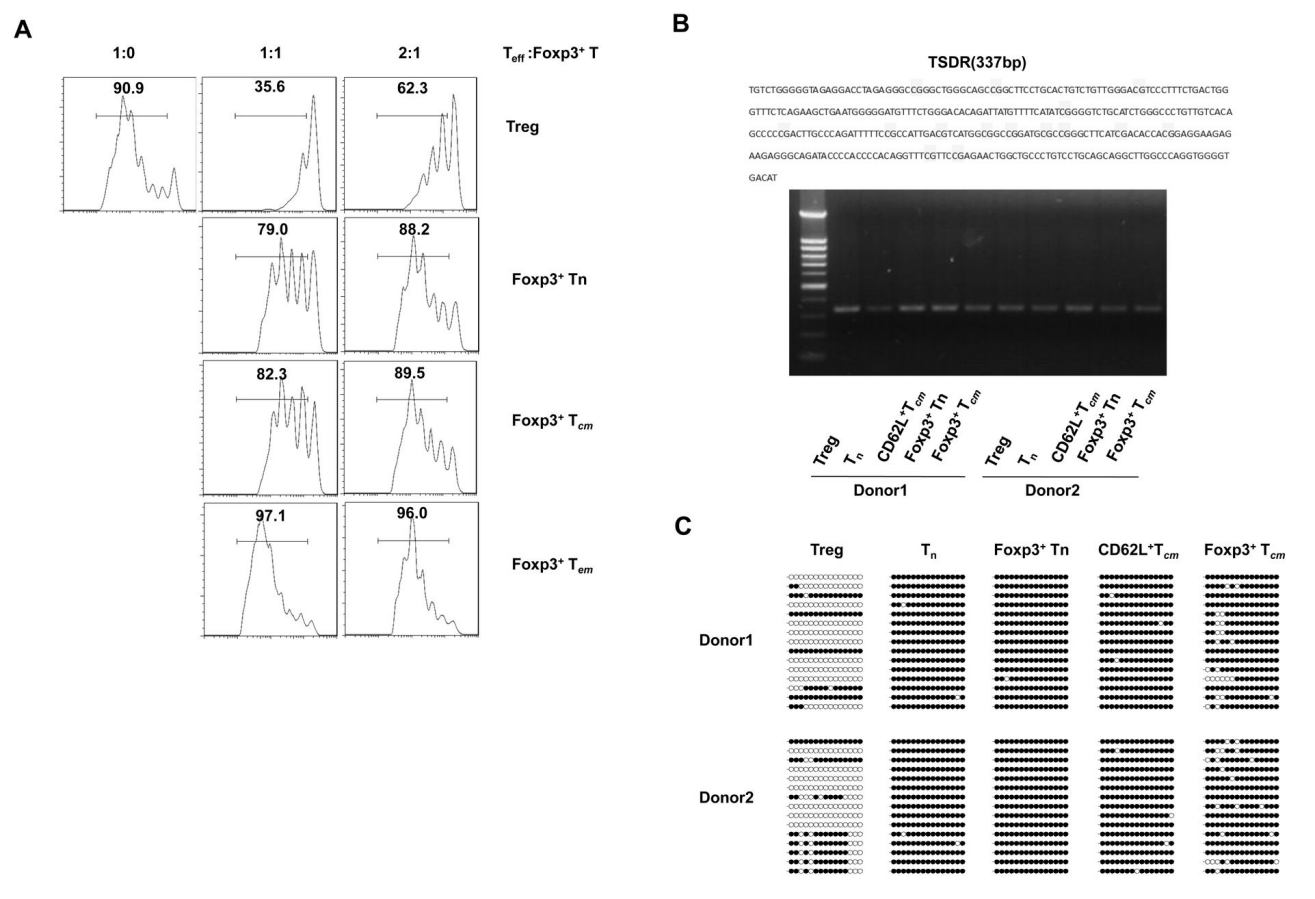
Suppressive functions and methylation status of Foxp3^+^ T cells derived from human CD4^+^CD62L^+^ central memory cells. ‘Treg’ indicates sorted CD4^+^CD25^high^ T cells, ‘Foxp3^+^ Tn’, ‘Foxp3^+^ T_*cm*_’ and ‘Foxp3^+^ T_*em*_’ indicate *in* *vitro* TGF-β-induced Foxp3^+^ cells from naive, CD62L^+^ T_*cm*_ and CD62L^-^ T_*em*_ CD4^+^ cells, respectively. (A) CFSE-labelled allogeneic CD4^+^CD25^-^ T cells (2.5×10^4^) cultured alone or mixed with different ratios of suppressor cells (1:0, 1:1 and 2:1) were activated with plate-bound anti-CD3 (5 μg/ml) and soluble anti-CD28 (1 μg/ml) in 96-well plates for 5 days. The suppressor cells were derived from naive, CD62^+^ T_*cm*_ and CD62L^+^ T_*em*_ cells that were induced to differentiate by the addition of TGF-β. The results are one representative of three independent experiments. (B) Electrophoresis of PCR (BSP) products sequenced with bisulphite. Treg indicates sorted CD4^+^CD25^high^ T cells, Tn indicates sorted CD4^+^ naive T cells, CD62L^+^ T_*cm*_ indicates sorted CD4^+^CD62L^+^ central memory T cells, Foxp3^+^ Tn indicates *in* *vitro* TGF-β-induced Foxp3^+^ cells from CD4^+^ naive T cells, and Foxp3^+^ T_*cm*_ indicates *in* *vitro* TGF-β-induced Foxp3^+^ cells from CD4^+^CD62L^+^ central memory T cells. (C) Analysis of FOXP3 TSDR methylation in cultured cells. Each line represents a clone, and every dot represents a CpG site in the FOXP TSDR. A solid black dot indicates a methylated CpG site, while a hollow dot indicates a demethylated CpG site.

We next examined whether Foxp3^+^ T cells derived from different subsets of human CD4^+^ T cells were functionally similar to Treg. We used induction procedures to differentiate Foxp3^+^ T cells from naive CD4^+^ T cells, CD62L^+^CCR7^+^ memory CD4^+^ T cells, and CD62L^-^CCR7^-^ memory CD4^+^ T cells before further evaluating their suppressive function by co-culture with varying ratios of responder CD4^+^CD25^-^ T cells. The CFSE proliferation study showed that the Foxp3^+^ T cells induced from the CD62L^+^CCR7^+^ memory cells did not exhibit the expected suppressive functions, as they were unable to suppress the responder CD4^+^CD25^-^ T cells even when stimulated at 1:1 (Foxp3^+^ T cells/T_eff_ cell) ratio (Figure 4A). The Foxp3^+^ T cells derived from naive CD4^+^ T cells displayed weak suppressive functions similar to the Foxp3^+^ T cells derived from CD62L^+^CCR7^+^ memory cells, which is consistent with a previous study showing that Foxp3^+^ T cells induced from naive CD4^+^ T cells by TGF-β and IL-2 treatment did not exhibit suppressive capabilities [12]. In addition, the Foxp3^+^ T cells induced from CD62L^-^CCR7^-^ memory CD4^+^ T cells did not exhibit any suppressive functions and even somewhat enhanced responder cell proliferation. We obtained the same results using another assay system in which APCs plus anti-CD3 were used stimulate the responder CD4^+^CD25^-^ T cells (Figure S3).

### Foxp3^+^ T cells derived from human CD4^+^CD62L^+^ central memory cells in vitro showed high methylation levels of FOXP3 TSDR

In Treg, the FOXP3 Treg-specific demethylated region (TSDR) is fully demethylated, and demethylation correlates with sustained Foxp3 expression. It was previously demonstrated that Foxp3^+^ T cells derived from human CD4^+^ naive T cells have poor suppressive functions, and this is likely because the TGF-β-mediated Foxp3 expression in these cells does not epigenetically alter the methylation status of the FOXP3 TSDR [20,27,28]. To examine whether the weak suppressive functions of the Foxp3^+^ T cells derived from human CD4^+^CD62L^+^ central memory cells correlated with a high methylation status of the FOXP3 TSDR, we induced Foxp3^+^ T cells from FACS-sorted human CD4^+^ naive and CD4^+^CD62L^+^ central memory cells *in vitro*. Sorted CD4^+^CD25^high^ Tregs, CD4^+^ naive and CD4^+^CD62L^+^ central memory cells were stimulated *in vitro* as controls. The methylation statuses of 15 CpG sites in the TSDR sequences were analysed (Figure 4B-C). The analysis revealed higher levels of TSDR methylation in the CD4^+^CD62L^+^ central memory cell-derived Foxp3^+^ T cells than in the sorted Tregs, although these sites had less TSDR methylation than CD4^+^ naive T cell-derived Foxp3^+^ T cells. The TSDR methylation status in the control experimental groups (sorted CD4^+^CD25^high^ Tregs, CD4^+^ naive and CD4^+^CD62L^+^ central memory cells) was consistent with a previous report [20].

### Mouse CD4^+^CD62L^+^ central memory T cells readily convert to functional Foxp3^+^ regulatory T cells

**Figure 5:**
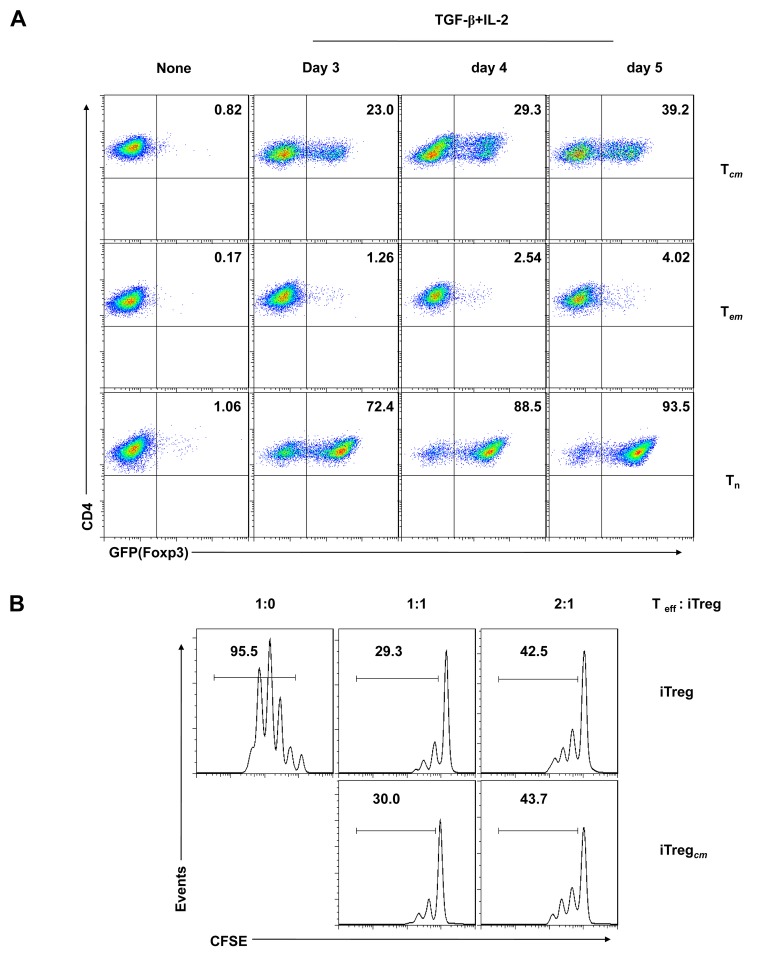
Induction of functional Foxp3^+^ T cells from mouse CD4^+^CD62L^+^ central memory T cells. (A) FACS-sorted mouse CD4 naive T cells (Tn), CD4 central memory T cells (T_*cm*_) and CD4 effector memory T cells (T_*em*_) were stimulated *in* *vitro* (1×10^5^ cells/well) with anti-CD3 (2 μg/ml) and APCs (1×10^5^ cells/well) in the presence of TGF-β (3 ng/ml) and IL-2 (100 U/ml) for 3–5 days. The induction of Foxp3 in the CD4^+^ fraction was analysed by FACS based on GFP expression. (B) CD4^+^ GFP^-^ (Foxp3^-^) T cells were sorted from Foxp3^gfp^ mice, labelled with CFSE and used as responder cells. The labelled T cells (1×10^5^ cells/well) were mixed with varying numbers of GFP^+^ (Foxp3^+^) T cells to achieve the desired Foxp3^+^ T cells/T effector cell ratios. GFP^+^ (Foxp3^+^) T cells were sorted from CD4^+^ naive or central memory T cells through the induction of Foxp3. ‘iTreg’ indicates *in* *vitro* TGF-β-induced GFP^+^ cells from CD4^+^ naive T cells, and ‘iTreg_*cm*_’ indicates *in* *vitro* TGF-β-induced GFP^+^ cells from CD4^+^CD62L^+^ central memory T cells. The cell mixture was stimulated with syngeneic APCs (1×10^5^ cells/well) and soluble anti-CD3 (1 μg/ml). Responder T cell proliferation with or without Foxp3^+^ T cells was assessed by CFSE dilution after 3 days of culture. The results are one representative of three independent experiments.

Foxp3^+^ T cells can be induced from murine naive CD4^+^ T cells by TGF-β and IL-2 and simultaneously obtain suppressive functions, which is different from human naive CD4^+^ T cells. This indicates that other unknown factors participate in the conversion of human Foxp3^-^ T cells to Foxp3^+^ iTreg [12]. We did not observe any suppressive activity of Foxp3^+^ T cells derived from CD4^+^CD62L^+^ central memory cells, likely for the same reason. Although it has been reported that mouse CD4^+^Foxp3^-^ effector memory T cells (CD44^high^CD62L^-^) are resistant to TGF-β-induced Foxp3 expression [10], it remains unknown whether mouse CD4^+^Foxp3^-^ central memory cells (CD44^high^CD62L^+^) exhibit the same property. To confirm this phenomenon, we sorted murine naive (CD44^low^CD62L^+^), central memory (CD44^high^CD62L^+^), and effector memory (CD44^high^CD62L^-^) cells from the CD4^+^GFP^-^ (Foxp3^-^) T cell pool of Foxp3^gfp^ mice by FACS. We then stimulated the cells *in vitro* with syngeneic APCs and soluble anti-CD3 in the presence of TGF-β and IL-2 and analysed the induction of Foxp3^+^ T cells by flow cytometry at different time points. As expected, mouse CD4^+^ naive T cells could be readily converted to Foxp3^+^ (GFP^+^) T cells (approximately 93%) after 5 days of culture. Similarly, the mouse CD4^+^ central memory T cells readily converted to Foxp3^+^ (GFP^+^) T cells (approximately 39%), although at a lower rate than the CD4^+^ naive T cells. In addition, CD4^+^ effector memory T cells were completely resistant to TGF-β-induced conversion, as previously reported (Figure 5A) [10]. To investigate the function of the induced Foxp3^+^ T cells, we first sorted GFP^+^ (Foxp3^+^) T cells by FACS within the pools of mouse CD4^+^ naive or central memory T cells. After returning them to culture, the CFSE-labelled responder T cells were mixed with varying numbers of sorted GFP^+^(Foxp3^+^) T cells to achieve the desired Foxp3^+^ T cells/T effector cell ratios. An analysis of responder T cell proliferation with Foxp3^+^ T cells showed that the Foxp3^+^ T cells derived from mouse CD4^+^ central memory T cells had clear suppressive function as compared to the CD4^+^ naive T cell-derived Foxp3^+^ T cells, they were really iTreg (Figure 5B).

## Discussion

Adaptive Treg cells (iTreg) are known to be involved in immune tolerance to foreign antigens and tumour-derived neo-antigens. Multiple reports have demonstrated roles for iTreg in mucosal immune tolerance, preventing the clearance of viruses and other microorganisms and obstructing the elimination of tumours [4,29-33]. However, the detailed mechanisms underlying their origin and differentiation are unknown. Although CD4^+^Foxp3^-^ naive T cells can be converted to iTreg by TGF-β, the ability of CD4^+^ memory T cells to differentiate into iTreg remains controversial. Some studies have reported that human skin-derived CD4^+^ memory T cells and mouse antigen-specific Th2 memory cells can be successfully converted to CD4^+^Foxp3^+^ T cells *in vitro* [14,15], and our data support this observation. 

In the peripheral blood of chronic HBV-infected patients, the percentage of CD4^+^Foxp3^+^ T cells was significantly higher than in healthy controls, and the increased numbers of CD4^+^Foxp3^+^ T cells displayed the CD45RO^+^ memory cell phenotype. In addition, although the *in vitro* differentiation efficiency of CD4^+^ memory T cells was significantly lower than that of the CD4^+^ naive T cells, there was a small subset of cells in the CD4^+^ memory T cell pool that could be converted to CD4^+^Foxp3^+^ T cells. These data, combined with those of previous reports, led us to hypothesise that a subset of CD4^+^ memory T cells might serve as the origin of some CD4^+^Foxp3^+^ T cells *in vivo*. In this study, we report a CD62L^+^CCR7^+^ subset of cells in the human CD4^+^ memory T cell pool that efficiently differentiated into CD4^+^Foxp3^+^ T cells by TCR stimulation with TGF-β and IL-2 *in vitro*. We purified the indicated T-cell subsets from human PBMCs and examined their *in vitro* differentiation efficiency by flow cytometry analysis. Although the CD4^+^ memory T cells had very low overall rates of differentiation into CD4^+^Foxp3^+^ T cells *in vitro*, the CD4^+^ central memory T cells had a slightly higher efficiency. We further identified a group of CD62L^+^ cells in the central memory pool with an *in vitro* differentiation efficiency that approached that of CD4^+^ naive T cells. However, an epigenetic analysis showed that the CD4^+^Foxp3^+^ T cells derived from CD62L^+^ central memory cells were more methylated at the FOXP3 TSDR compared to the sorted Treg, which could explain why these CD4^+^Foxp3^+^ T cells did not exhibit suppressive functions. Additionally, we confirmed that mouse CD62L^+^ central memory cells could readily convert to Foxp3^+^ iTreg, and those iTreg had clear suppressive functions. These new findings suggest that a particular subset of CD4^+^ memory T cells, defined as the CD62L^+^ central memory cells, have the capacity to convert to CD4^+^Foxp3^+^ T cells.

In the case of HBV, higher levels of Treg have been described [34]. It is well known that not all human Foxp3^+^ cells are Treg, particularly under conditions of chronic cell activation, such as a viral infection, where there may be a higher proportion of effector T cells that transiently upregulate Foxp3 [35,36]. However, the frequency of total CD4^+^CD25^+^ T cells was not significantly different between chronic HBV-infected patients and healthy controls [37]. In addition, HBV-infected patients in the inactive (IN) phase have low HBV DNA levels (<2000 IU/ml) and normal ALT levels with minimal or absent hepatic inflammation [38,39]. This indicates that CD4^+^ T cells are not activated in chronic HBV-infected patients in the inactive (IN) phase. Thus, the incremental CD4^+^Foxp3^+^ T cells in the PBMCs of chronic HBV-infected patients in our study can be considered to be induced Foxp3^+^ T cells. The increased number of CD45RO^+^CD4^+^Foxp3^+^ T cells in chronic HBV-infected patients may represent HBV-induced CD4^+^Foxp3^+^ T cells because the CD4^+^ T cells were not activated *in vivo*.

Because Foxp3 expression is not induced during the activation of mouse CD4^+^CD25^-^ non-regulatory T cells, Foxp3 is considered to be the definitive marker of mouse nTreg, iTreg generated in the periphery in vivo, and iTreg generated in vitro in the presence of TGF-β [40]. In contrast, several studies have claimed that this is not the case in humans. They suggest that human CD4^+^CD25^-^ T effector cells activated through TCR stimulation alone with anti-CD3 and anti-CD28 show transiently upregulated Foxp3 expression and demonstrate that this Foxp3 expression correlates with neither anergy nor suppressive function [35,41-45]. In addition to confirming these previous studies, our results shown in Figure 2A and Figure 3A reveal new aspects involving Foxp3 expression. Consistent with previous studies, upon stimulation with anti-CD3 and anti-CD28, the memory CD4^+^CD25^-^ T cells in our study transiently upregulated their expression of Foxp3, and a dramatic increase in the percentage of Foxp3^+^ cells was observed, with up to 18% of the memory CD4^+^ T cells expressing Foxp3 on day 3 (Figure 2A). The proportion of Foxp3^+^ T cells then progressively diminished over time to 7% by day 7. Interestingly, we found that naive CD4^+^CD25^-^ T cells did not always transiently upregulate Foxp3 expression upon TCR stimulation alone with anti-CD3 and anti-CD28. Previous studies based on human CD4^+^CD25^-^ T cells did not reveal this phenomenon, i.e., that the transient upregulation of Foxp3 expression through TCR stimulation alone without TGF-β is restricted to memory CD4^+^CD25^-^ T cells. Furthermore, memory (Tm), central memory (T_*cm*_), and effector memory (T_*em*_) CD4^+^CD25^-^ T cells stimulated in the presence of TGF-β also showed an increase in Foxp3 expression compared with those stimulated without TGF-β (Figure 2A and Figure 3A). Although the effect of TGF-β was not as strong in naive CD4 CD4^+^CD25^-^ T cells, these results still suggest that some memory CD4^+^CD25^-^ T cells respond to TGF-β-mediated Foxp3 expression.

Previous results have shown that the methylation status of the FOXP3 TSDR is associated with the development of stable Treg properties, and rare demethylation has been observed in activated human conventional CD4^+^ T cells or in CD4^+^ naive T cell-derived CD4^+^Foxp3^+^ T cells [20,27,46-48]. Our current data clearly show that TGF-β-induced CD4^+^Foxp3^+^ T cells derived from CD62L^+^ central memory cells exhibited high methylation of CpG sites in the FOXP3 TSDR sequence. These results may explain why the CD4^+^Foxp3^+^ T cells derived from the CD62L^+^ central memory cells did not exhibit clear suppressive functions (Figure 4A). It remains unclear why the FOXP3 TSDR was not demethylated in these CD4^+^Foxp3^+^ T cells, but we can speculate that the demethylation of the FOXP3 TSDR during the formation of Treg is accomplished through a far more complicated epigenomic regulatory network and involves the coordination of multiple extracellular signals.

Our data indicate that CD62L^+^ central memory cells can efficiently differentiate into CD4^+^Foxp3^+^ T cells. However, the detailed mechanism of this differentiation is still unclear and requires further study. We hypothesise that it is related to the intrinsic constraints of the cells but not due to their similarity to the CD4^+^ naive T-cell phenotype. Human CD4^+^ memory T cells are not a uniform population, and they can be divided into the two functionally distinct subsets of CCR7^+^ central memory and CCR7^-^ effector memory T cells [49]. There are significant differences between these two subsets of cells, as illustrated by their heterogeneity and effector functions, among other factors [50,51]. Of note, CD4^+^ central memory and effector memory cells have different capacities to produce cytokines. Upon activation, CD4^+^ effector memory cells secrete high levels of IL-4 and IFN-γ, which are not produced by activated CD4^+^ central memory cells or CD4^+^ naive T cells [49]. Additionally, it is widely accepted that IL-4 and IFN-γ are the major cytokines involved in the inhibition of TGF-β-induced Foxp3 expression in CD4^+^CD25^-^ T cells [9-11,52]. Therefore, differences in cytokine secretion could potentially explain why CD4^+^ central memory T cells can better differentiate into Foxp3^+^ T cells.

In conclusion, we propose for the first time that CD4^+^CD62L^+^ central memory T cells can be induced to express Foxp3 by TGF-β both in human and mouse, and obtained functional Foxp3^+^ iTreg from mouse CD4^+^CD62L^+^ central memory T cells. Our data suggested the possibility that iTreg might be derived from activated CD4^+^CD62L^+^ central memory T cells *in vivo*, at least in mouse. Thus, our study provides novel information on the source of iTreg. Unfortunately, it seemed likely that we failed to obtain functional Foxp3^+^ T cells from human CD4^+^CD62L^+^ central memory T cells in this study. But considering the induced Treg cells used for the assay were not purified FOXP3 expression T cells, they were a mixed population of Foxp3^+^ T cells and activated T effector cells, insufficient FOXP3 expression T cells added might lead to the results we observed, their suppressive functions might be obscured by mixed activated T effector cells. Our further research confirmed this, induced Foxp3^+^ cells from naive and CD62L^+^ central memory cells exhibited some suppressive functions when the ratio (T_*eff*_ cells/Foxp3^+^ T cells) was increased (data not show), though their suppressive functions were not apparent compared to that of Tregs directly isolated from PBMCs. These findings suggested that such human induced Foxp3^+^ cells might have suppressive functions. We will further explore the suppressive functions of human Foxp3^+^ cells in our ongoing research.

## Supporting Information

Figure S1 **Expression of CD45RA in the samples shown in Figure 1E.** Naive CD4^+^ T cells were sorted from adult peripheral blood using FACS, and the cells were activated with plate-bound anti-CD3 (5 μg/ml) and soluble anti-CD28 (1 μg/ml) in the absence or presence of TGF-β (5 ng/ml) and IL-2 (100 UI/ml) for 5 days. The cells were then collected and analysed by flow cytometry to evaluate the expression of Foxp3 and CD45RA.
(TIF)

Figure S2 **Expression of CD45RA and CD45RO in human CD4^+^ T cells.** Analysis of the CD45RA and CD45RO expression on freshly isolated PBMCs FACS gated for CD4^+^ T cells.
(TIF)

Figure S3 **Suppressive functions of Foxp3^+^ T cells derived from human CD4^+^CD62L^+^ central memory cells.** ‘Treg’ indicates sorted CD4^+^CD25^high^ T cells, ‘Foxp3^+^ Tn’, ‘Foxp3^+^ T_*cm*_’ and ‘Foxp3^+^ T_*em*_’ indicate *in* *vitro* TGF-β-induced Foxp3^+^ cells from naive, CD62L^+^ T_*cm*_ and CD62L^-^ T_*em*_ CD4^+^ cells, respectively. CFSE-labelled allogeneic CD4^+^CD25^-^ T cells (2.5×10^4^ cells/well) cultured alone or mixed with different ratios of suppressor cells (1:0, 1:1 and 2:1) were activated with anti-CD3 (5 μg/ml) and APCs (5×10^4^ cells/well) in 96-well plates for 5 days. The suppressor cells were derived from naive, CD62^+^ T_*cm*_ and CD62L^+^ T_*em*_ cells that were induced to differentiate by the addition of TGF-β.
(TIF)

Table S1 **Information for the human samples.** Detailed information regarding the human samples used in our study, including age and sex.
(TIF)
