# Supplementary figures and images for: CD4+CD62L+ Central Memory T Cells Can Be Converted to Foxp3+ T Cells
Source: PLoS One. 2013 Oct 14;8(10):e77322. doi: 10.1371/journal.pone.0077322 (PMC3796486; doi:10.1371/journal.pone.0077322)

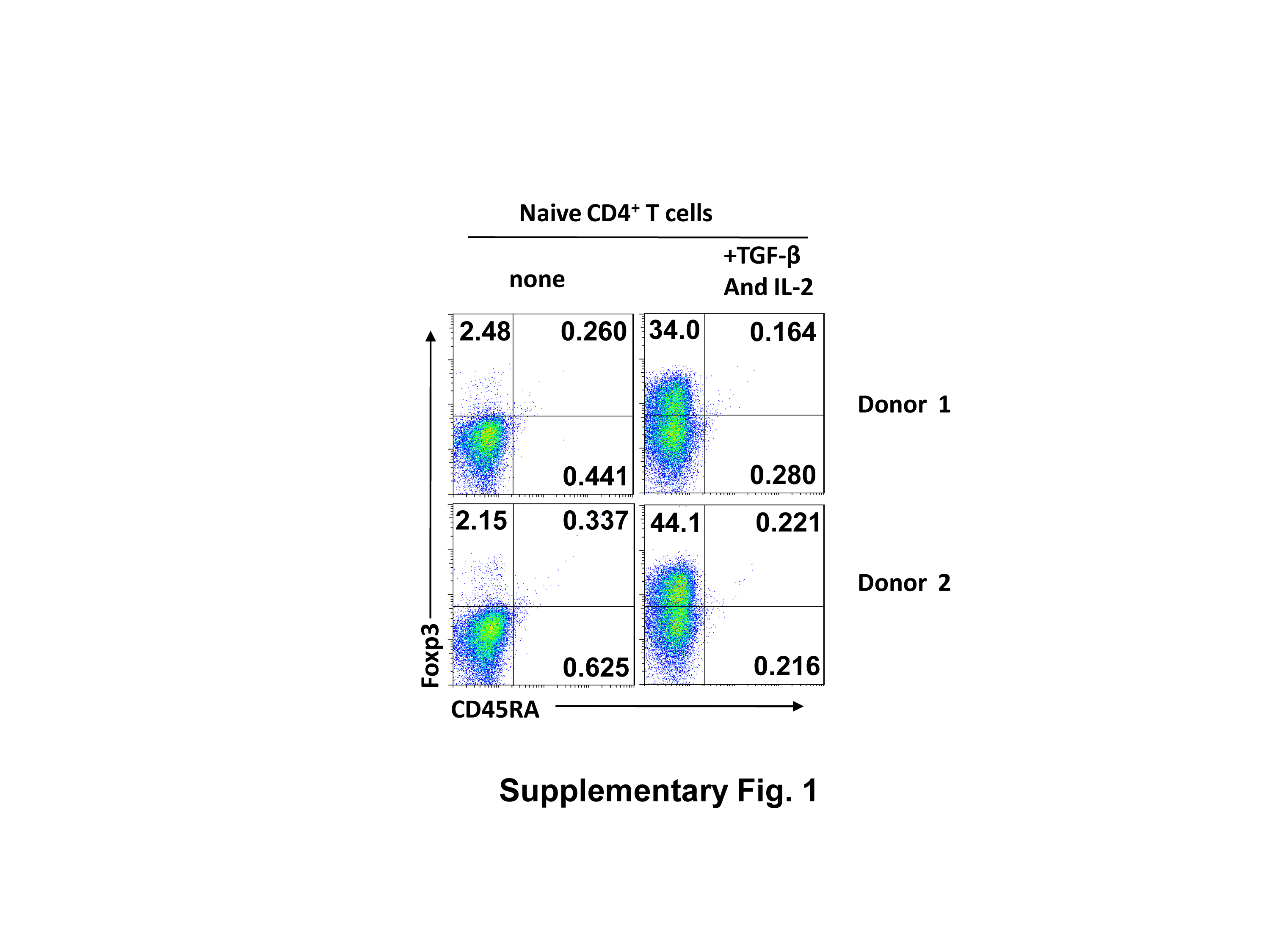

Supplement: Figure S1 — Expression of CD45RA in the samples shown in Figure 1E. Naive CD4+ T cells were sorted from adult peripheral blood using FACS, and the cells were activated with plate-bound anti-CD3 (5 μg/ml) and soluble anti-CD28 (1 μg/ml) in the absence or presence of TGF-β (5 ng/ml) and IL-2 (100 UI/ml) for 5 days. The cells were then collected and analysed by flow cytometry to evaluate the expression of Foxp3 and CD45RA. (TIF) [file pone.0077322.s001.tif]

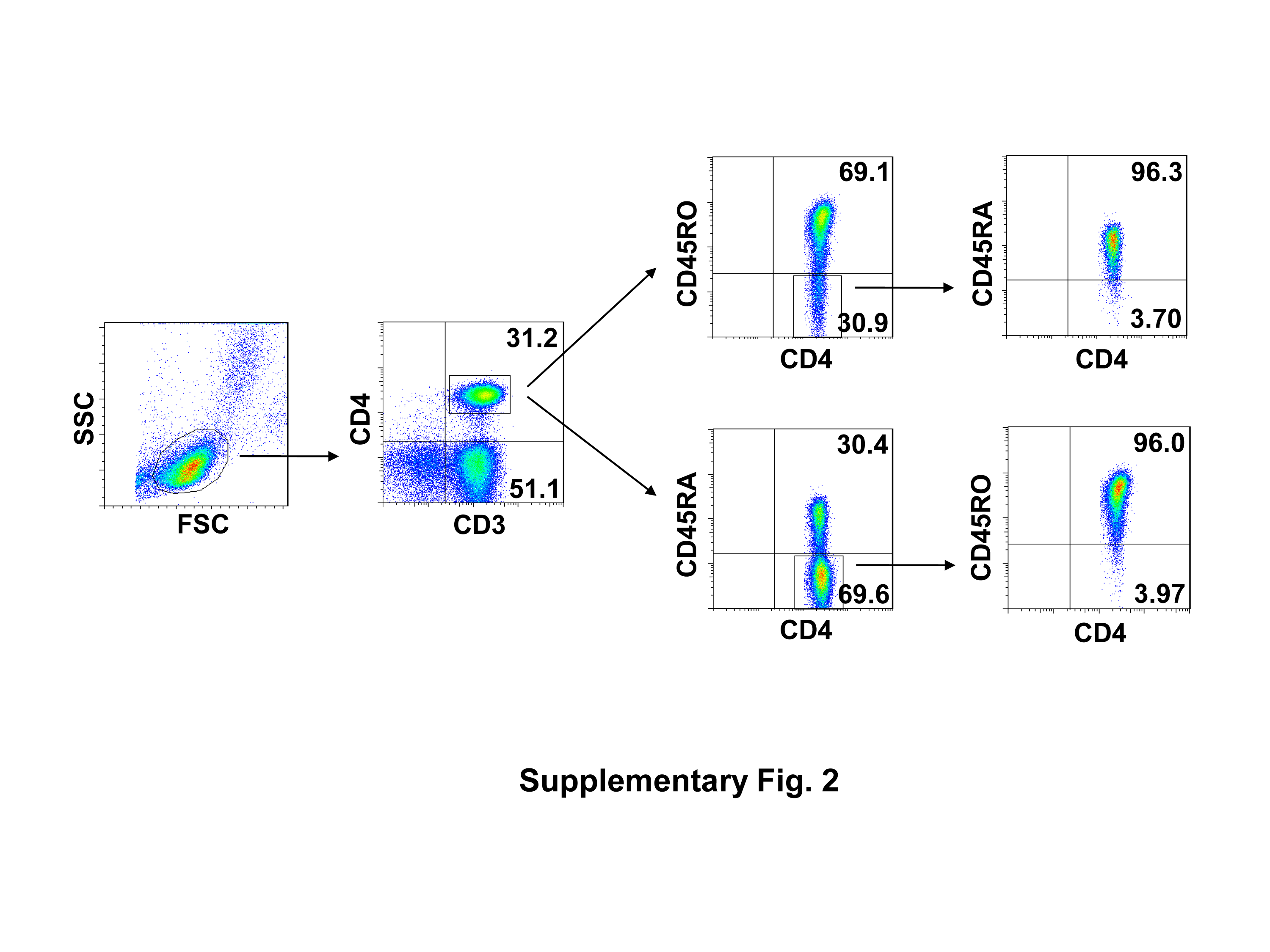

Supplement: Figure S2 — Expression of CD45RA and CD45RO in human CD4+ T cells. Analysis of the CD45RA and CD45RO expression on freshly isolated PBMCs FACS gated for CD4+ T cells. (TIF) [file pone.0077322.s002.tif]

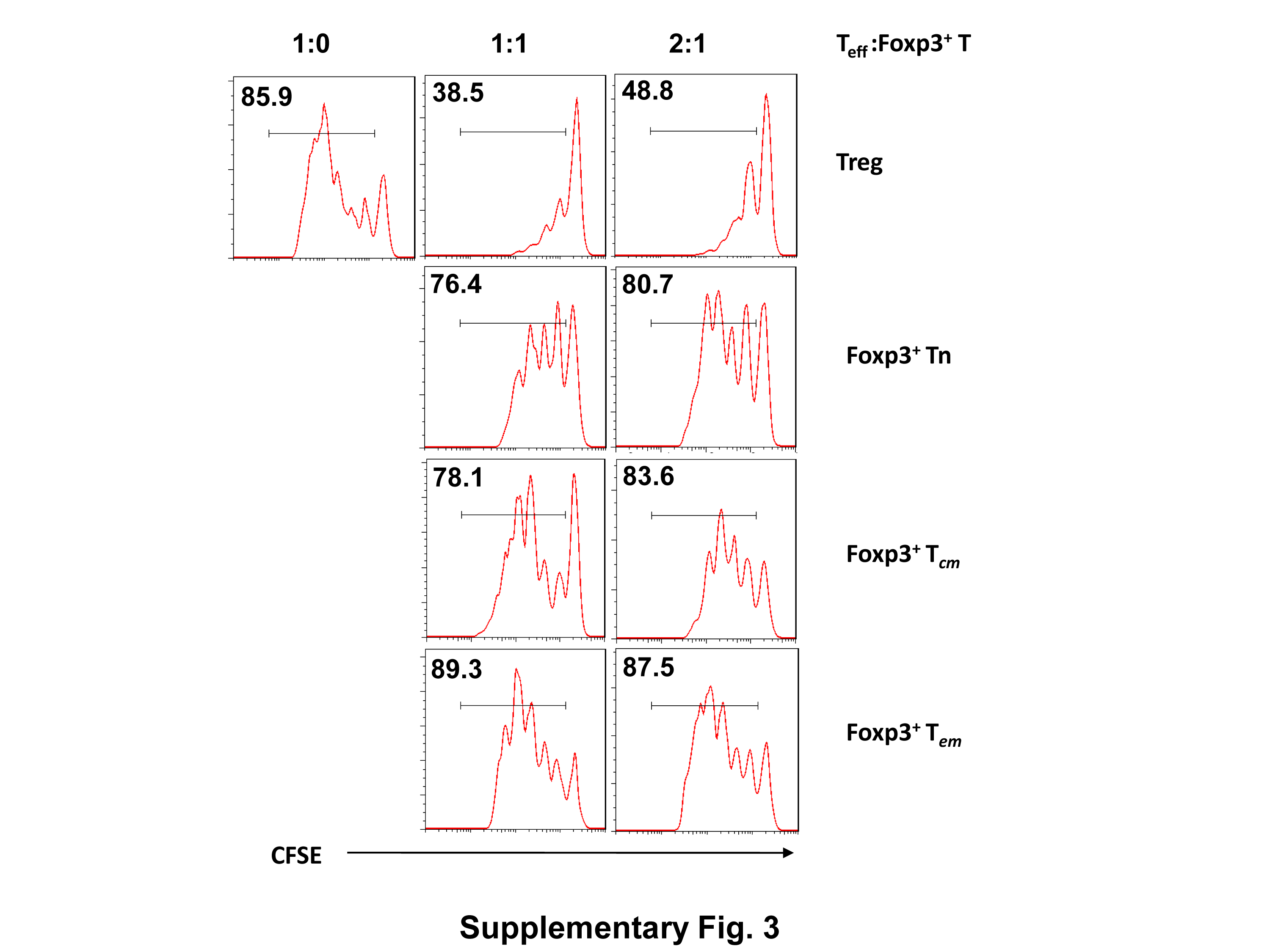

Supplement: Figure S3 — Suppressive functions of Foxp3+ T cells derived from human CD4+CD62L+ central memory cells. ‘Treg’ indicates sorted CD4+CD25high T cells, ‘Foxp3+ Tn’, ‘Foxp3+ Tcm’ and ‘Foxp3+ Tem’ indicate in vitro TGF-β-induced Foxp3+ cells from naive, CD62L+ Tcm and CD62L- Tem CD4+ cells, respectively. CFSE-labelled allogeneic CD4+CD25- T cells (2.5×10^4^ cells/well) cultured alone or mixed with different ratios of suppressor cells (1:0, 1:1 and 2:1) were activated with anti-CD3 (5 μg/ml) and APCs (5×10^4^ cells/well) in 96-well plates for 5 days. The suppressor cells were derived from naive, CD62+ Tcm and CD62L+ Tem cells that were induced to differentiate by the addition of TGF-β. (TIF) [file pone.0077322.s003.tif]

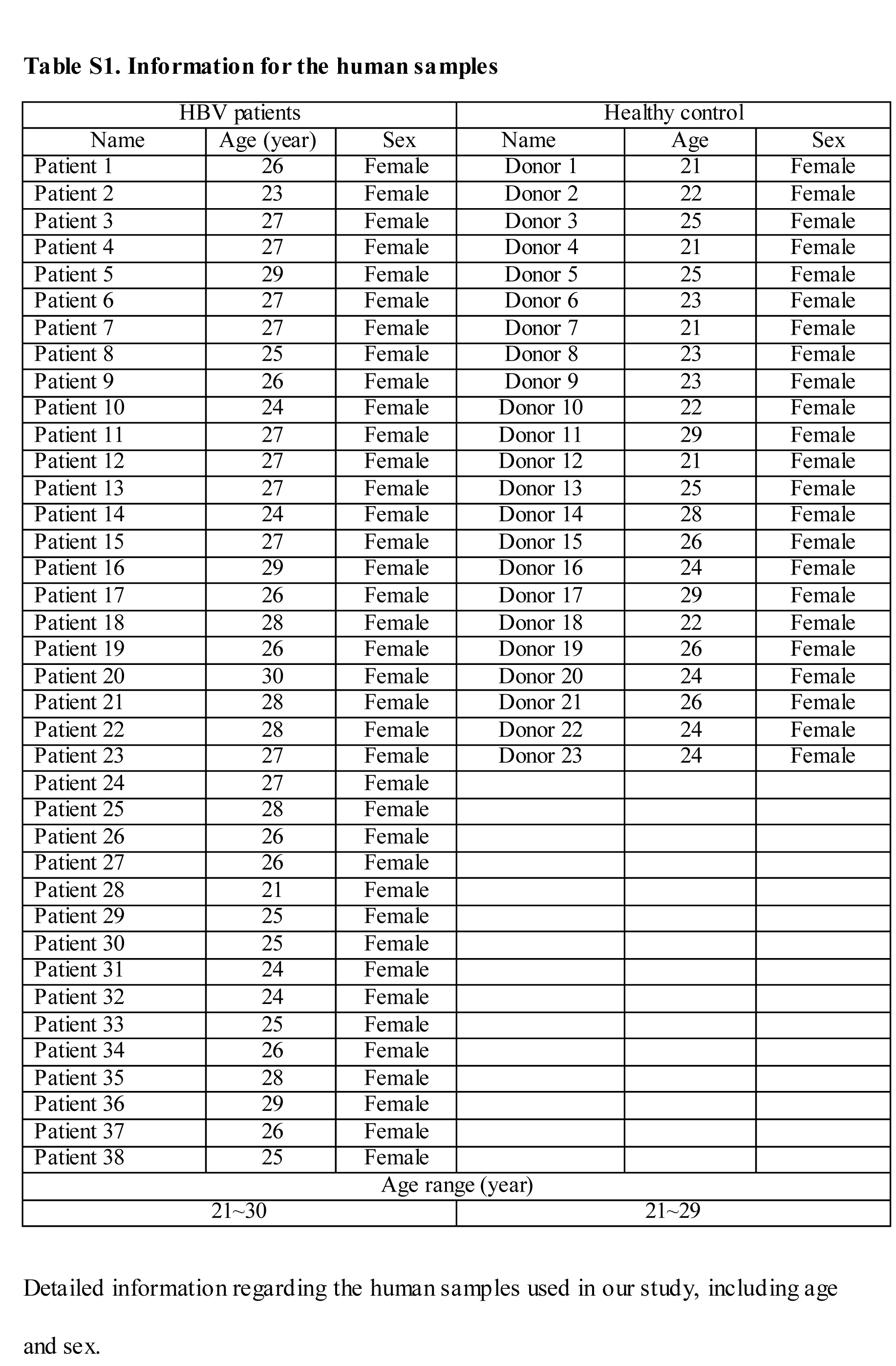

Supplement: Table S1 — Information for the human samples. Detailed information regarding the human samples used in our study, including age and sex. (TIF) [file pone.0077322.s004.tif]
